# Sleep Apnea and Abnormal Respiratory Patterns with Deep Sedation during Radiofrequency Catheter Ablation in Patients with Atrial Fibrillation

**DOI:** 10.31083/j.rcm2304121

**Published:** 2022-04-01

**Authors:** Yasuhiro Tomita, Yuka Kimura, Satoshi Kasagi, Takatoshi Kasai

**Affiliations:** ^1^Sleep Center, Toranomon Hospital, 105-8470 Tokyo, Japan; ^2^Cardiovascular Center, Toranomon Hospital, 105-8470 Tokyo, Japan; ^3^Department of Cardiovascular Medicine, Juntendo University Graduate School of Medicine, 113-8421 Tokyo, Japan; ^4^Cardiovascular Respiratory Sleep Medicine, Juntendo University Graduate School of Medicine, 113-8421 Tokyo, Japan; ^5^Okinaka Memorial Institute for Medical Research, 105-8470 Tokyo, Japan

**Keywords:** obstructive sleep apnea, central sleep apnea, atrial fibrillation, radiofrequency catheter ablation, sedation

## Abstract

**Background:**

Abnormal respiration during radiofrequency catheter ablation (RFCA) with deep sedation in patients with atrial fibrillation (AF) can affect the procedure’s success. However, the respiratory pattern during RFCA with deep sedation remains unclear. This study aimed to investigate abnormal respiration during RFCA and its relationship with sleep apnea in patients with AF.

**Methods:**

We included patients with AF who underwent RFCA with cardiorespiratory monitoring using a portable polygraph both at night and during RFCA with deep sedation. The patients were divided based on the administered sedative medicines.

**Results:**

We included 40 patients with AF. An overnight sleep study revealed that 27 patients had sleep apnea; among them, 9 showed central predominance. During RFCA with deep sedation, 15 patients showed an abnormal respiratory pattern, with 14 patients showing obstructive predominance. Further, 17 and 23 patients were administered with propofol alone and dexmedetomidine plus propofol, respectively. There was no significant between-group difference in the respiratory event index (REI) at night (7.9 vs. 9.3, *p* = 0.744). However, compared with the group that received dexmedetomidine plus propofol, the propofol-alone group showed a higher REI during RFCA (5.4 vs. 2.6, *p* = 0.048), more frequent use of the airway (47% vs. 13%, *p* = 0.030), and a higher dose of administered propofol (3.9 mg/h/kg vs. 1.2 mg/h/kg, *p *< 0.001). Multivariable analysis revealed that only the propofol amount was associated with REI during RFCA (*p* = 0.007).

**Conclusions:**

Our findings demonstrated that respiratory events during RFCA with deep sedation were mainly obstructive. Propofol should be administered with dexmedetomidine rather than alone to reduce the propofol amount and avoid respiratory instability.

## 1. Introduction

Atrial fibrillation (AF) is often related to sleep apnea [1] and there is a need to elucidate their relationship. AF is a common disease that is recently frequently treated with radiofrequency catheter ablation (RFCA) therapy. The widespread use of ablation therapy could be attributed to improvements in treatment techniques and sedative use, which have shortened the treatment time, improved the success rate, and reduced complications [2]. Moreover, the presence of sleep apnea in patients with AF is associated with the success rate of ablation therapy [3]. Continuous positive airway pressure (CPAP) therapy is the gold standard for obstructive apnea; further, it reduces AF recurrence after ablation [4, 5].

Obesity and apnea are associated with the success rate of ablation [6]. The sudden cessation and resumption of breathing during ablation with sedation could prolong the treatment time and reduce the success rate. In Japan, some electrophysiology cardiologists prefer using adaptive servo-ventilation (ASV) to stabilize breathing during ablation [7].

Central and obstructive apneas are common sleep-related breathing disorders related to AF; however, there have been inconsistent reports regarding the frequency of central apnea [8]. The occurrence of central apnea during ablation compared with that during sleep, as well as the significance of using ASV during ablation, remain unclear. This study aimed to investigate sleep apnea in patients with AF undergoing ablation therapy and respiratory abnormalities during ablation with sedatives.

## 2. Materials and Methods

### 2.1 Study Population

We included consecutive patients who were admitted to our hospital for RFCA from January 2016 to May 2017. We excluded patients aged <20 years, patients already treated for sleep apnea, patients with uncompensated heart failure, and patients who had previously undergone cardiac surgery. This study was approved by the Toranomon Hospital Ethics Committee (No.1273). All the included participants provided informed consent.

### 2.2 Data Collection

Upon admission for ablation therapy, height and weight measurements, as well as responses to the Epworth Sleepiness Scale (ESS) questionnaire, were collected. For echocardiography, we used data obtained within six months before ablation therapy.

The patients underwent cardiorespiratory monitoring using a portable digital polygraph (SAS-3200, Nihon Kohden, Tokyo, Japan) during the night before RFCA and during the RFCA procedure with deep sedation. Arterial oxygen saturation (${\mathrm{SaO}}_{2}$) and the heart rate were recorded. Oronasal airflow was monitored using a nasal cannula; additionally, thermocouple signals and snoring were monitored using a nasal pressure transducer. Chest wall and abdominal movements were monitored using piezo respiratory effort sensors. Respiratory events were scored on the preceding night and during the RFCA based on the American Academy of Sleep Medicine scoring manual. Hypopnea was scored using desaturation according to the home sleep apnea testing rules for adults in the manual version 2.2. Additionally, we recorded the self-reported monitoring time of the overnight study.

The monitoring time during ablation was defined as the time from sedative administration to the end of the ablation procedure unless the following situations occurred: oxygen administration; mechanical airway clearance; and other operations that interrupted cardiopulmonary recordings, including pericardiocentesis for cardiac tamponade. The respiratory event index (REI) was defined as the number of events during the monitoring time of each setting. We defined sleep apnea as a nighttime REI ≥5; additionally, abnormal breathing during ablation was defined according to the REI value calculated based on the number of events and the monitoring time during ablation.

### 2.3 RFCA with Deep Sedation

Before procedure commencement, cardiorespiratory monitoring equipment, as well as monitors required for the procedure, were placed. This was followed by the administration of sedatives; specifically, propofol alone or in combination with dexmedetomidine, to achieve deep sedation and reduce intraprocedural movement. The operator preoperatively determined the sedative choice. Concomitant administration of dexmedetomidine began after August 2016. Further, the operator who performed ablation operator was blinded to the presence or absence of apnea at night. In case of use of the nasopharyngeal airway during the ablation or starting of supplemental oxygen, respiratory events were calculated until the time for analysis.

### 2.4 Statistical Analysis

Continuous variables are expressed as mean and standard deviation or median and interquartile range, while categorical variables are presented as numbers and percentages.

Two groups of patients were compared based on the presence or absence of sleep apnea and sedative medication. Between-group comparisons of categorical and continuous variables were performed using the chi-squared test or Fisher’s exact test and the *t*-test or Mann-Whitney U-test, respectively. A multivariate regression model was used to identify predictors of REI values during ablation. In this model, the explanatory variables included age, sex, body mass index (BMI), REI value during the day, and propofol administration rate. Statistical analyses were performed using R software, version 3.4.3 (R Core Team, Vienna, Austria). Two-sided *p* values < 0.05 were considered statistically significant.

## 3. Results

We included 40 patients (85% men; mean age: 60 years; mean BMI: 23.7). The median time since AF diagnosis was 18 months; further, 30% of the patients had chronic AF and the median ESS score was 6 points. In the nocturnal sleep study, the mean recording time was 435 minutes. The median REI was 9.0. Further, 27 patients had sleep apnea, with 18 and 9 patients showing obstructive and central respiratory events, respectively. There were no significant differences in age, sex, and BMI between patients with or without apnea (cut-off REI value of 5/h). There was no significant difference in the median ESS score between patients with and without apnea (5.5 vs. 9.0, *p* = 0.743). Additionally, there were no significant between-group differences in the rate of chronicity and duration of AF. Data obtained from echocardiography, including the left ventricular ejection fraction and left atrial diameter, were not significantly associated with the presence of sleep apnea (Table 1).

**Table 1.** **Patient characteristics**.

	Sleep apnea (n = 27)	No sleep apnea (n = 13)	*p* value
Age, years	61 ± 11	57 ± 12	0.331
Male, n (%)	25 (93%)	9 (69%)	0.053
BMI, kg/$m^{2}$	24.0 ± 3.1	23.1 ± 3.7	0.485
ESS	5.5 (3.0–7.8)	9.0 (4.5–12.0)	0.743
Persistent AF, n (%)	18 (67%)	10 (77%)	0.507
History of AF, months	16 (4.5–66)	22 (6.0–72)	0.675
LVEF, %	64.4 ± 5.9	67.7 ± 8.0	0.196
LAD, mm	39.9 ± 6.2	36.4 ± 5.4	0.073

BMI, body mass index; ESS, Epworth Sleepiness Scale; AF, atrial fibrillation; LVEF, left ventricular ejection fraction; LAD, left atrial dimension.

Comparison according to the sedatives used during ablation revealed that the combination group was significantly older than the propofol alone group. However, there were no significant between-group differences in other background factors, nighttime REI values, and event type (obstructive or central) (Table 2). Regarding cardiorespiratory monitoring during ablation, the mean recording time was 173 minutes and the median REI was 3.5. Additionally, 15 patients developed abnormal breathing during ablation, with 14 and 1 patient showing obstructive and central respiratory events, respectively. Compared with the combination group, the propofol-alone group showed significantly higher REI values and use of the nasopharyngeal airway. There was no between-group difference in supplemental oxygen use. Contrastingly, the combination group showed a significantly higher propofol administration rate than the propofol-alone group (Table 3).

**Table 2.** **Sleep study**.

	Propofol-alone group (n = 17)	Combination group (n = 23)	*p* value
Age, years	55 ± 10	63 ± 10	0.016
Male, n (%)	15 (88%)	19 (83%)	0.999
BMI, kg/$m^{2}$	24.0 ± 3.5	23.4 ± 3.1	0.594
ESS	5 (3.5–5.5)	6 (3–8.8)	0.180
Persistent AF, n (%)	4 (24%)	8 (35%)	0.505
History of AF, months	24 (8–72)	13 (4–62.5)	0.933
LVEF, %	65.8 ± 7.6	65.2 ± 6.2	0.789
LAD, mm	38.9 ± 7.1	38.7 ± 5.5	0.929
Recording time, min	493 ± 93	431 ± 87	0.797
REI, /h	7.9 (4.0–13.3)	9.3 (2.7–12.4)	0.744
Lowest ${\mathrm{SaO}}_{2}$, %	88% (86–93%)	90% (85–91%)	0.865

BMI, body mass index; ESS, Epworth Sleepiness Scale; AF, atrial fibrillation; LVEF, left ventricular ejection fraction; LAD, left atrial dimension; REI, respiratory index; ${\mathrm{SaO}}_{2}$, arterial oxygen saturation.

**Table 3.** **Cardiorespiratory monitoring during ablation**.

	Propofol-alone group (n = 17)	Combination group (n = 23)	*p* value
Recording time, min	135 ± 96	200 ± 76	0.028
REI, /h	5.4 (2.0–11.4)	2.6 (1.5–4.8)	0.048
Lowest ${\mathrm{SaO}}_{2}$, %	88% (84–90%)	90% (87–95%)	0.347
Supplemental oxygen	17 (100%)	19 (83%)	0.123
Nasopharyngeal airway	8 (47%)	3 (13%)	0.030
Propofol, mg/h/kg	3.9 ± 1.7	1.2 ± 0.5	<0.001

REI, respiratory index; ${\mathrm{SaO}}_{2}$, arterial oxygen saturation.

**Fig. 1.**
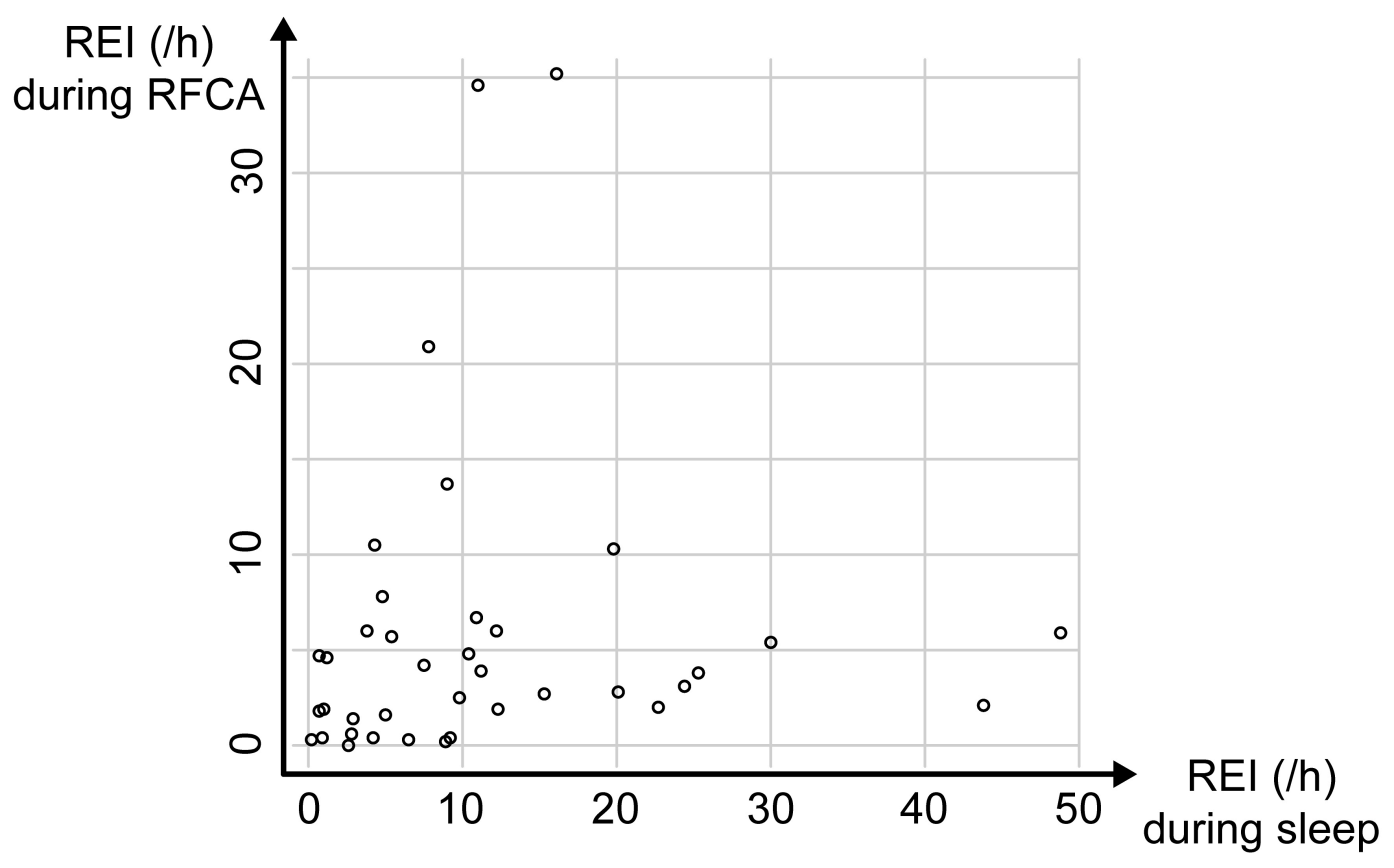
**Relationship between the respiratory event index (REI) during sleep and the REI during ablation**. There was no significant correlation between nighttime REI and REI during radiofrequency catheter ablation (RFCA).

There was no significant correlation between the nighttime REI and the REI during ablation (r = 0.06, *p* = 0.689, Fig. 1). The multivariate model revealed that only the rate of propofol administration was a significant predictor of REI during ablation (Table 4).

**Table 4.** **Multivariable linear regression analysis of REI during ablation**.

	β	*p* value
Age	0.07	0.586
Sex	1.92	0.571
BMI	0.01	0.993
REI during sleep	0.07	0.579
Propofol	2.6	0.002

BMI, body mass index; REI, respiratory index; β, standard partial regression coefficient.

## 4. Discussion

In our study, among 40 participants scheduled for AF ablation, two-thirds had sleep apnea; among them, one-third showed predominantly central sleep apnea (CSA). Moreover, approximately one-third of the patients with respiratory events scored as apneas or hypopneas under sedation during ablation, with most being obstructive events. Nocturnal REI was not a predictor of the severity of apneic events during ablation; instead, the amount of propofol used for sedation was a significant predictor.

AF is associated with a high incidence of apnea (21%–74%) [1]. However, few studies have described the association of AF with obstructive and central apnea separately. AF is an established risk factor for CSA, which is considered independent of the presence or absence of HF. However, the rate of CSA complications and their pathogeneses remain unclear [8]. In our study, one-third of the patients with nocturnal apneas showed predominantly central apneas. Future studies on the predictive factors for the development of central events are warranted. It remains unclear whether CPAP therapy for apnea in patients with AF who underwent ablation reduces AF recurrence. However, it is known that patients with AF have poor adherence to CPAP, which could be partly attributed to the fact that they are not sleepy even though they have sleep apnea [9]. In our study, approximately one-third of the patients with AF had CSA, which may result in poor adherence to CPAP treatment. We believe that evaluation of residual apnea and, if necessary, use of ASV may be a treatment option.

Propofol or midazolam may be used for deep sedation during ablation therapy; furthermore, respiratory support, including airway or mask-supported positive pressure breathing, may be required [7]. Moreover, dexmedetomidine is a sedative with low respiratory depression that has been used for non-intubated patients requiring sedation for surgical and diagnostic procedures [10]. We found that respiratory events, especially obstructive events, appeared during sedation in a manner dependent on the propofol dose. Since the propofol amount can be reduced by the concomitant use of dexmedetomidine, this combination strategy can allow decreased respiratory events during sedation.

Several studies have shown that ASV is helpful as a respiratory aid during ablation procedures for AF. However, in our study, the respiratory events during ablation were mainly obstructive; therefore, CPAP may be acceptable rather than ASV. It may be desirable to use a nasal airway or similar device to avoid obstruction. Since many patients present with central apnea at night, periodic breathing after block release should be considered.

This study has several limitations. First, we performed cardiorespiratory monitoring using a portable monitor rather than full polysomnography. We used the same device at night and during the ablation in order to obtain similar parameters for both periods. Therefore, hypopnea could only be scored for events with desaturation, which may result in underestimation. Nevertheless, obstructive and central events were differentiated by monitoring the respiratory effort and snoring sounds. Second, recordings during ablation were short (mean ≈3 h) while the nighttime recordings were adequate (mean >7 h). One case in the propofol and combination groups each showed a recording during ablation of <30 minutes; however, the results were similar even after the exclusion of these cases from the analysis. Third, there was a tendency to use dexmedetomidine mainly in elderly patients. The operator independently made the decision to combine propofol with dexmedetomidine while blinded to the presence or absence of nocturnal sleep apnea. Finally, given the small sample size in our study, the findings of the multivariate analysis must be interpreted with caution. Although there were significant between-group differences in the REI values during ablation, the power was insufficient for detection.

## 5. Conclusions

In conclusion, AF-related sleep apnea was frequent; additionally, one-third of the patients with sleep apnea predominantly showed central events. Given the variety of AF-related respiratory events, appropriate evaluation is necessary before interventions for sleep apnea in patients with AF.
